# Supplementary material for: Learning curve and surgical outcome of robotic assisted colorectal surgery with ERAS program
Source: Sci Rep. 2022 Nov 29;12:20566. doi: 10.1038/s41598-022-24665-w (PMC9709162; doi:10.1038/s41598-022-24665-w)
Supplement: Supplementary file 2 — Supplementary Information 2. [file 41598_2022_24665_MOESM2_ESM.zip › RAL Raw data-Ñ[▒K/050/HS_ñw╝╨░O▒Kñσ.pdf]

正式摘要(電子簽章：000000208055639)		
W52 / W52 ☐英文標題(注意:無英文姓名-請補病人基本檔!)	☐【讀取】憑證	

臺中榮民總醫院

出院病歷摘要

轉入號：　　轉出號：

[redacted]

**(8)**流水編號	
**(9)**入院日期	110/01/08 (2021) 直腸外科 W52 - 161
**(10)**轉科日期	
**(11)**出院日期	110/01/14 (2021) 住院天數計 6 日

診斷

- 入院：1. Sigmoid colon cancer with multiple metastasis
  2. hypertension
  3. type 2 diabete mellitus
- 出院：1. Sigmoid colon cancer with multiple metastasis s/p robot assisted low anterior resection on 2021/1/9
  2. hypertension
  3. type 2 diabete mellitus

主訴

- intermittent abdominal pain for about half year

病史

- This 46 years old male patient has past history of hypertension, type 2 diabete mellitus. He complained intermittent abdominal pain that relieved after defecation. He also noticed that stool became thinner with blood-tinged. Body weight decreased about 8 kg in this 6 months. He came to CMUH for help. Colonoscopy was done that sigmoid colon tumor was seen biopsy showed malignancy. Abdominal CT was done that multiple hepatic tumor was seen. Sigmoid colon cancer with multiple metastasis was impressed. He came to our hospital for help. Obstruction was found in colonoscopy. Thus, he was admitted for surgical treatment.

體檢發現

- Anal inspection:
  Recto digital examination:
  Sigmoidoscopy:
  Anoscopy:
  Abdomen:
  - General appearance :
    - easy looking , conscious clear , without cardiopulmonary distress
    - Vital signs自動帶入date 體溫(BT) 脈搏(P) 呼吸(R) 血壓(BP) 身高(BH) 體重(BW) BMI
      20210108 36℃ 101次/分 20次/分 122/76mmHg 167.9cm 77kg 27.3
  - HEENT :
    - Eyes: no anemic conjunctiva, no icteric sclera, normal EOM
    - ENT: no otorrhea, no rhinorrhea, no erythematous mucosa, no injected tonsil, no deformity
  - Neck: supple, freely movable, trachea midline,no deformity, no engorged jugular vein , no palpable thyroid or lymph node enlargement
  - Chest:
    - Inspection: Grossly normal, symmetric and fully expansion, no accessory muscle used, no operation scar, no spider angiomata, no gynecomastia
    - Palpation: no palpable mass or nodule, no subcutaneous emphysema, no tenderness
    - Ausculation: clear breathing sound
  - Heart:

-
  - Percussion: normal heart size
  - Ausculation: regular heart beat, normal S1, S2, no gallop, no murmur
- Abdomen:
  - Inspection: in
    - flat contour, symetrically, no scar, no stria, no dilated veins, no rashes
  - Ausculation: normoactive bowel sound, no abdominal bruits
  - palpation: soft, no muscle guarding or rigidity, no local tenderness, no rebounding pain, no palpable mass, no palpable pulsation
  - Percussion: no shifting dulllness, no hepatomegaly, no splenomagaly
-

Peripheral pulsation :	CA	BA	RA	FA	PA	PTA	DPA
Right side :	++	++	++	++	++	++	++/+++
Left side :	++	++	++	++	++	++	++/+++

- Genito-anus: no tenderness or palpable mass lesion or rectal shelf, normal external genitalia, normal anal tone, brown stool

CRS CONDITION

- CEA: 73.22 ng/ml
-
  - Lung

手術日期及方法

- 手術日期及方法20210109 1. Robotic (XI) assisted anterior resection
  2. Intraoperative colonoscopy : (-).
  3. High ligation (+) : At IMA root. Left colic artery and IMV were also ligated via Robotic endoclips.
  4. Take down from the splenic flexure : (-)
  5. Robotic endoGIA (45-3.8) X1 + EEA (29mm) were applied
  6. Proximal cut end: 8 cm, distal cut end: 3 cm
  7. ADHESION BARRIER FILM was placed below the central abdominal wound

住院治療經過

- After admission, robot assisted low anterior resection was done on 1/9 as scheduled. After operation we let him try water and then diet as ERAS protocol. Under the stable condition, he was discharged on 1/14.

合併症

- nil

一般檢查

- 累積報告 :

DATE	TIME	ALB	TP	BIL.T	BIL.D	ALKP	AST	ALT	LDH	CHOL	TG	HDL-C	LDL	UA	NH3	AMY
1100109	1441	3.5		0.33			17	11								

DATE	TIME	WBC	RBC	HGB	HCT	MCV	PLT	BLST	NEUT	BAND	SEG	LYM	MON	EOS	BAS
1100109	1441	5790		9.3	31.2		250		91.7			6.7	1.4	0.0	0.2

DATE	TIME	NA	K	CL	CA	BUN	CREAT	Mg	P	CRP	ESR	PCT	LIPASE	LACTATE
1100109	1441	141	4.2		8.1	10	0.91							

特殊檢查

病理報告

其它

出院時情況

- 3.改門診治療

出院指示

-

藥名	劑量	劑量單位	用法	頻次	數量	數量單位	現況	天數	單位
Xanax tab 0.5mg	1	TAB	PO	TID	15	TAB	TH	5	天

$Biofermin tab	2	TAB	PO	TID	30	TAB	TH	5	天
Flurbiprofen 100mg	1	TAB	PO	BID	10	TAB	TH	5	天
Silence tab 1mg	1	TAB	PO	QN	5	TAB	TH	5	天
Mag. Oxide tab	2	TAB	PO	TID	30	TAB	TH	5	天
Ulstop tab 20mg	1	TAB	PO	BID	10	TAB	TH	5	天

出院計劃

主治醫師_2062F 蔣鋒帆 / 住院醫師_2331F 陳凱祥

正式摘要(電子簽章：**000000208055639)**

W52 / W52

doctorRank:B
